# Penetration of MeV electrons into the mesosphere accompanying pulsating aurorae

**DOI:** 10.1038/s41598-021-92611-3

**Published:** 2021-07-13

**Authors:** Y. Miyoshi, K. Hosokawa, S. Kurita, S.-I. Oyama, Y. Ogawa, S. Saito, I. Shinohara, A. Kero, E. Turunen, P. T. Verronen, S. Kasahara, S. Yokota, T. Mitani, T. Takashima, N. Higashio, Y. Kasahara, S. Matsuda, F. Tsuchiya, A. Kumamoto, A. Matsuoka, T. Hori, K. Keika, M. Shoji, M. Teramoto, S. Imajo, C. Jun, S. Nakamura

**Affiliations:** 1 Institute for Space-Earth Environmental Research, Nagoya University, Nagoya, 464-8601 Japan; 2 Graduate School of Communication Engineering and Informatics, University of Electro-Communications, Chofu, 182-8585 Japan; 3 Research Institute for Sustainable Humanosphere, Kyoto University, Uji, 611-0011 Japan; 4 National Institute of Polar Research, Tachikawa, 190-8518 Japan; 5 University of Oulu, Pentti Kaiteran katu 1, Linnanmaa, Oulu, Finland; 6 National Institute of Information and Communications Technology, Tokyo, 184-8795 Japan; 7 Japan Aerospace Exploration Agency (JAXA), Sagamihara, 252-5210 Japan; 8 Sodankylä Geophysical Observatory, University of Oulu, Sodankylä, Finland; 9 Space and Earth Observation Centre, Finnish Meteorological Institute, Helsinki, Finland; 10 Graduate School of Science, University of Tokyo, Tokyo, 113-0033 Japan; 11 Graduate School of Science, Osaka University, Toyonaka, 560-0043 Japan; 12 Graduate School of Natural Science and Technology, Kanazawa University, Kanazawa, 920-1192 Japan; 13 Graduate School of Science, Tohoku University, Sendai, 980-8578 Japan; 14 Graduate School of Science, Kyoto University, Kyoto, 606-8502 Japan; 15 Graduate School of Engineering, Kyushu Institute of Technology, Fukuoka, 820-8501 Japan; 16 The Graduate University for Advanced Studies, SOKENDAI, Hayama, 240-0193 Japan; 17 Joint Support-Center for Data Science Research, Research Organization of Information and Systems, Tachikawa, 190-8518 Japan

**Keywords:** Aurora, Atmospheric chemistry

## Abstract

Pulsating aurorae (PsA) are caused by the intermittent precipitations of magnetospheric electrons (energies of a few keV to a few tens of keV) through wave-particle interactions, thereby depositing most of their energy at altitudes ~ 100 km. However, the maximum energy of precipitated electrons and its impacts on the atmosphere are unknown. Herein, we report unique observations by the European Incoherent Scatter (EISCAT) radar showing electron precipitations ranging from a few hundred keV to a few MeV during a PsA associated with a weak geomagnetic storm. Simultaneously, the Arase spacecraft has observed intense whistler-mode chorus waves at the conjugate location along magnetic field lines. A computer simulation based on the EISCAT observations shows immediate catalytic ozone depletion at the mesospheric altitudes. Since PsA occurs frequently, often in daily basis, and extends its impact over large MLT areas, we anticipate that the PsA possesses a significant forcing to the mesospheric ozone chemistry in high latitudes through high energy electron precipitations. Therefore, the generation of PsA results in the depletion of mesospheric ozone through high-energy electron precipitations caused by whistler-mode chorus waves, which are similar to the well-known effect due to solar energetic protons triggered by solar flares.

## Introduction

Visible light from aurorae is emitted by the excitation of neutral atmospheric components^1^*.* The precipitation of magnetospheric electrons and their acceleration along the magnetic field lines is the primary mechanism responsible for discrete aurorae, which are seen as curtain-like structures from the ground. On the other hand, diffuse aurorae, which may not be easily visible from the ground, are caused by the precipitation of charged particles (principally electrons) without field-aligned accelerations. It is assumed that the precipitation of diffuse auroral electrons is caused by pitch-angle scattering through plasma wave interactions in the magnetosphere. Typical diffuse aurora mainly results from precipitation of ~ 1 keV electrons^2^. Pulsating aurorae (PsA) are a relatively faint class of diffuse aurorae that exhibits quasi-periodic luminosity modulations^3,4^. Sounding rocket and low-altitude spacecraft observations have shown that these modulations are associated with intermittent electron precipitation ranging from a few keV to a few tens of keV^4–9^.

The Van Allen radiation belt possesses the highest energy charged particle population in geospace, and relativistic electrons are trapped in the outer Van Allen radiation belt^10,11^. Precipitations of relativistic electron of the outer Van Allen radiation belt have been observed, which are categorized into different groups^12,13^. Relativistic electron microbursts^14^ that are spikes of high precipitation flux on subsecond timescales are observed. Recently, it has been suggested that relativistic electron microbursts occur during PsA and relativistic electrons precipitate into the upper and middle atmosphere^15^. The precipitating relativistic electrons can reach altitudes below the typical aurora emission altitudes, resulting in mesospheric ionization and the consequent depletion of ozone molecule O_3_ at mesospheric altitudes. However, previous conjugate satellite and ground-based optical observations^15^ have not detected such low-altitude ionization, so that the maximum energy of precipitating electrons is not known. The presence of high-energy electrons in PsA can only be inferred from direct observations of the electron density by incoherent scatter radars in the mesosphere in concert with ground-based optical imager and satellite observations. In the present study, we report unique coordinated experiments for the observation of PsA in Scandinavia, which were realized by the European Incoherent Scatter (EISCAT) radar^16^*,* the all-sky camera network, and the Japanese spacecraft Arase^17^. The data are useful for estimating the possible ozone destruction due to relativistic electron precipitations associated with PsA.

## Arase and EISCAT observations

**Figure 1:**
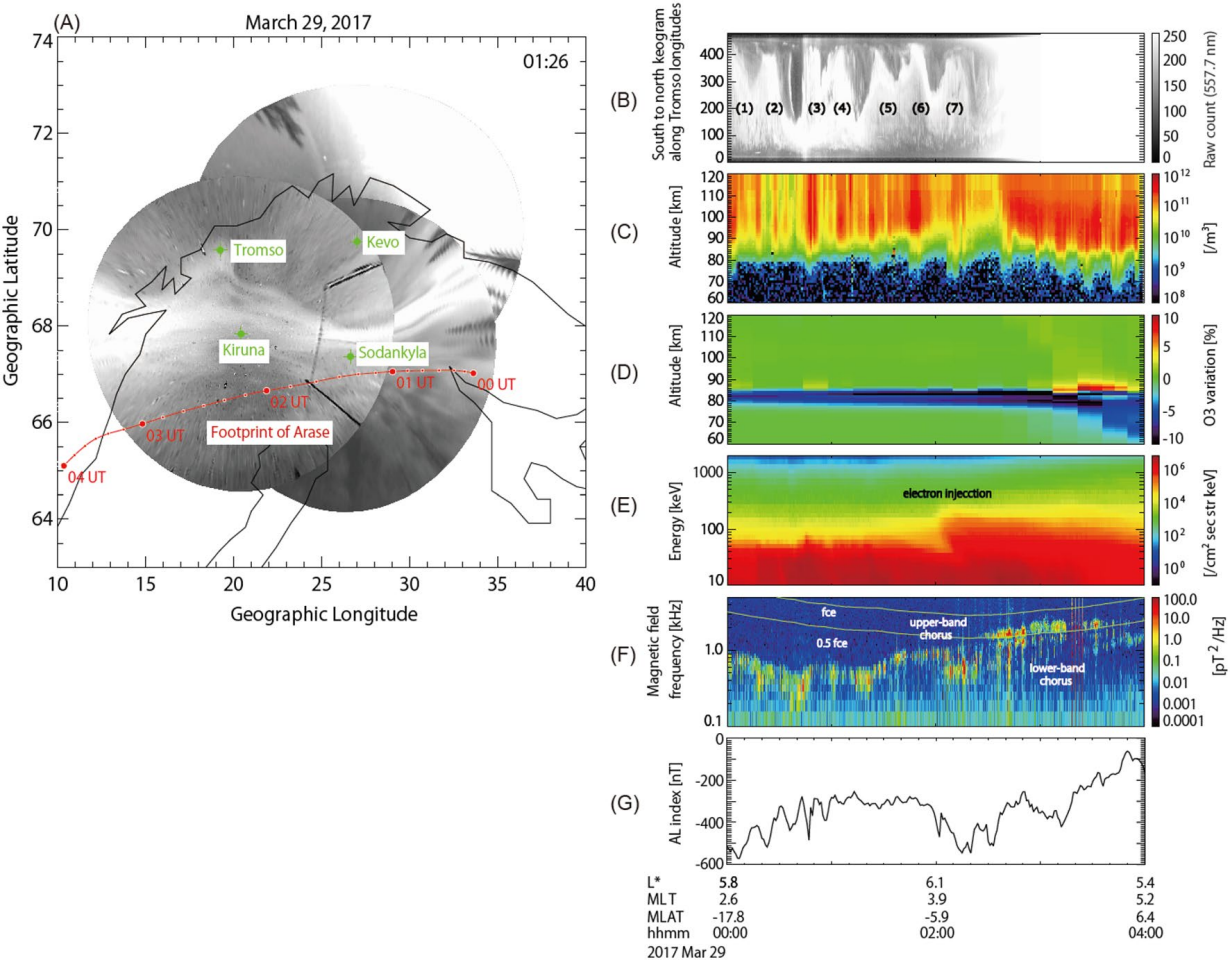
Geospace conditions from the upper atmosphere to the magnetosphere. (**A**) Map around the Scandinavian peninsula with the footprint of Arase from 00:00 UTC to 04:00 UTC on March 29, 2017. Aurora images were obtained from the all sky imager network. (**B**) Keograms of an aurora image along the longitudes of Tromsø, Norway. Labels (1–7) show the “omega-band” structures. (**C**) Electron density profile from a vertical beam of the EISCAT VHF radar at Tromsø, Norway, on March 29, 2017. The horizontal axis represents the universal time; the vertical axis denotes the altitude. The colour bar indicates the electron density. (**D**) Relative O_3_ profile from the computer simulation based on the EISCAT observations. The colour bar indicates relative variations from the controlled run without energetic electron precipitations. (**E**) Energy-time diagram of electrons measured by the MEP-e/HEP/XEP instruments onboard the Arase spacecraft. The horizontal axis represents the universal time; the vertical axis denotes the electron energy. Here, MEP-e, HEP, and XEP denote the medium-energy particle—electron, high-energy particle, and extremely high-energy particle, respectively. The colour bar indicates the differential flux of electrons. (**F**) Frequency-time diagram of the magnetic field components of plasma waves measured by the Arase spacecraft. The vertical axis denotes the plasma wave frequency. The colour bar indicates the power spectrum density of the waves. Two lines correspond to the electron gyrofrequency ($f_{{ce}}$) and their hall frequency ($0.5f_{e}$). (**G**) AL index. (IDL ver.8.7, https://www.l3harrisgeospatial.com/Software-Technology/IDL).

At the end of March 2017, a high-speed solar wind stream arrived at Earth causing moderate geomagnetic storms for a few days. Figure 1A illustrates the footprints of Arase mapped along the field line and presents aurora images obtained from the imager network. The Arase footprints traversed the Scandinavia Peninsula from 00:00 to 04:30 UTC on March 29, 2017. The aurora image is a snapshot at 01:27 UTC, and wave-like structures, known as the “omega-band” aurora signature, are seen at 68°–70° latitudes. Figure 1G shows the AL index, from which several substorm activities are identified. The EISCAT radar at Tromsø, Norway, observed the ionospheric electron density profile at altitudes between 60 and 120 km, which are directly related to the energy spectrum of precipitating electrons. The Arase spacecraft observed electrons and ions as well as electric and magnetic fields and waves in the Van Allen radiation belts.

Figures 1B–F illustrates a series of data obtained from the ground (aurora), ionosphere (electron precipitation), mesospheric ozone simulation, and magnetosphere. Figure 1B shows an aurora keogram at the Tromsø longitude during the event period. A series of omega-band structures is developed every ~ 30 min; seven omega-bands were identified in total, as shown in Fig. 1B. The vertical stripes appearing over a wide latitudinal range manifest the appearance of PsA. Figure 1C presents the temporal variation of the height profile of the electron density. The electron density enhancements occurred intermittently at altitudes below 70 km. It is noteworthy that the electron density enhancements are seen at around 65 km after 03:00 UTC. The altitude of 65 km is one of the lowest observed ionization altitudes associated with PsA.

**Figure 2:**
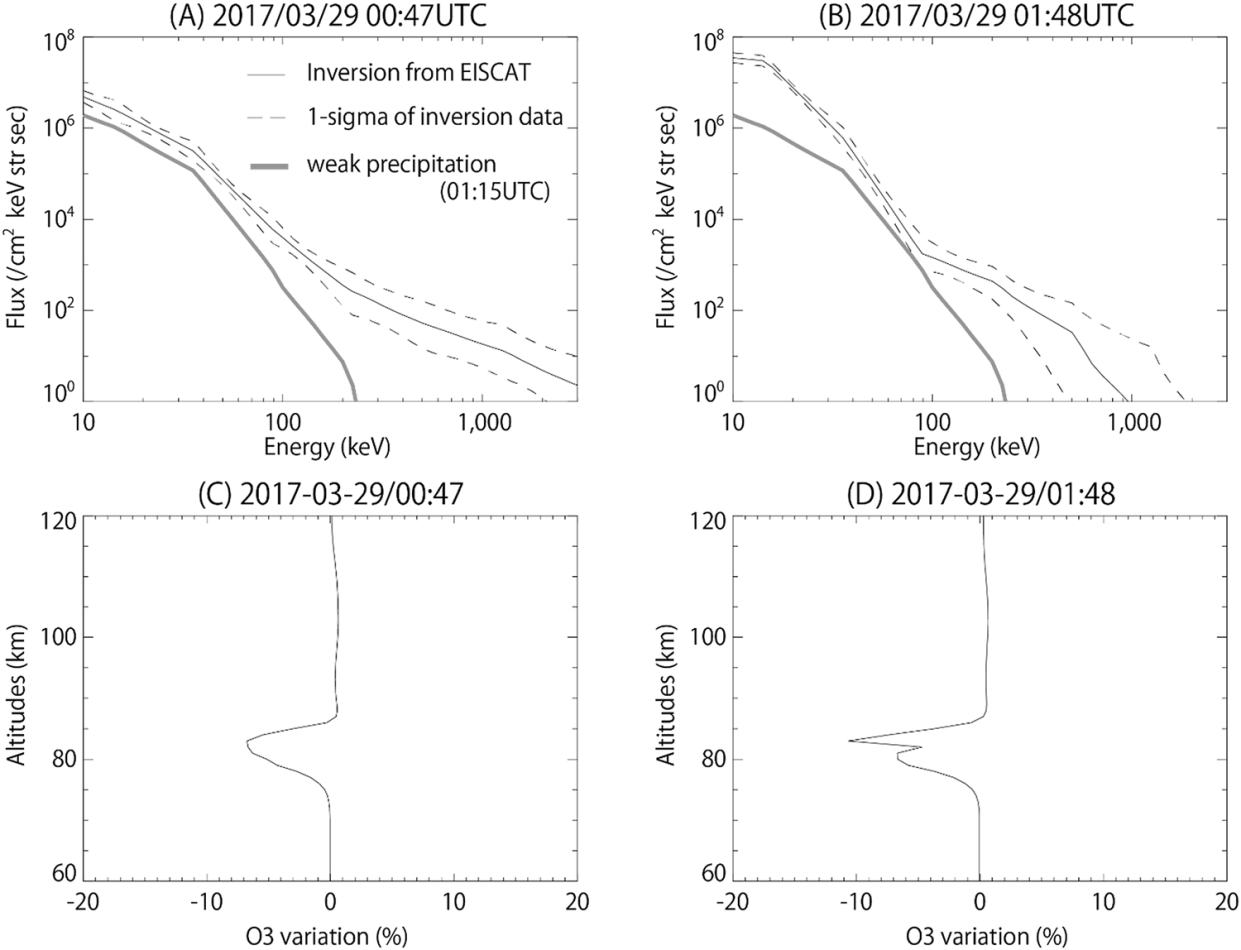
Energy spectrum of precipitating electrons and ozone depression. (**A**,**B**) Estimated energy spectrum obtained from EISCAT measurements using the inversion method^12^. The black dashed line indicates the 1-sigma errors. Thick line is a reference of weak electron precipitation observed at 01:15UTC on March 29, 2017. (**C**,**D**) Altitude profile of O_3_ variation. The profiles mean the relative difference to the control run without MeV electron precipitations.

The energy spectrum of precipitating electrons is derived by an inversion calculation using the height profile of electron density^18^. Figure 2A,B present the estimated energy spectrum of precipitating electrons obtained from the EISCAT observations at the selected time interval. Figure 2A,B represent the spectra at 0:47 and 1:48 UTC, respectively, when the omega-band structures traversed above Tromsø, as shown at (3) and (6) in Fig. 1B. By comparing the time variations of the energy spectrum and keogram, we deduce that MeV electron precipitations occur in association with PsA embedded in the omega-bands, indicating that MeV electron precipitations seemingly correlate with the repeated development of the omega-bands. The maximum energy of precipitating electrons exceeds 2 MeV.

The ionization induced by such precipitations may cause chemical consequences, especially in the concentration of odd nitrogen (NO_x_ = N + NO + NO_2_) via the dissociation of molecular nitrogen and odd hydrogen (HO_x_ = H + OH + HO_2_) due to ion-pair production^19^, which can catalytically deplete mesospheric odd oxygen (O_x_ = O + O_3_)^18^. This scenario has been verified by computer simulations, including a comprehensive description of the ion chemistry at altitudes between 20 and 150 km^18^.

Figure 1D presents the temporal variation of the height profile of O_3_ concentration predicted by the computer simulation, Sodankylä Ion and Neutral Chemistry Model (SIC)^20^. The figure shows the relative variations of O_3_ between the cases with and without (control run) electron forcing based on the EISCAT measurements^16^. Above 80 km, catalytic ozone depletion is inefficient due to lack of HOx production. On the other hand, the catalytic reaction sequences that cause ozone depletion require atomic oxygen which at night is abundant in the upper mesosphere only. Thus before 03:00 UTC, more than 10% O_3_ depletion is predicted but around 80 km altitudes only, although strong precipitation is observed, as shown in Fig. 1C. After 03:00 UTC, solar UV radiation increases production of atomic oxygen throughout the mesosphere after the sunrise, and catalytic O_3_ depletion extends down to 60 km altitudes. Note that the short-term enhancement around 85 km is due to a combination of PsA-driven atomic oxygen production and lack of catalytic loss. Figure 2C,D show the height profile of O_3_ concentration at 0:47 and 1:48 UTC, respectively. The simulation results confirm that the largest O_3_ depletion of 10% is observed during the PsA. In particular, electron precipitation associated with PsA makes a dominant role in the production of NO_x_ and HO_x_, which leads to O_3_ depletion in the mesosphere.

Figure 1E shows the energy spectra of electrons trapped in the magnetosphere with energies from 7 keV to 3 MeV as a function of time; the data are acquired from multi-instrument measurements onboard the Arase spacecraft^17,21–24^. During the observation period, the Arase spacecraft observed the trapped MeV electrons of the outer Van Allen radiation belt. At around 02:00 UTC, a flux enhancement of electrons is observed above 100 keV followed by subsequent enhancements of electron fluxes of tens of keV. This flux enhancement is referred to as electron injection, i.e., fresh electrons from the night-side plasma sheet enter the inner magnetosphere. These electrons are responsible for generating whistler-mode chorus waves^25^*.*

Figure 1F shows the wave power of plasma waves as a function of the frequency and time observed by the Arase spacecraft^17^ during the above-mentioned period. Intense lower-band chorus (LBC) and upper-band chorus (UBC) waves are recorded below and above half the electron-gyrofrequency, respectively. The average amplitudes of the LBC and UBC waves at 02:30 UTC, when the Arase was located near the magnetic equator, are 60 pT and 80 pT, respectively, which are typical chorus wave amplitudes during PsA^7^. The ambient plasma density is ~ 0.6 cm^−3^, which is estimated from the frequency of the upper-hybrid resonance waves^26^ and the ambient magnetic field^24^ measured by the Arase spacecraft.

**Figure 3:**
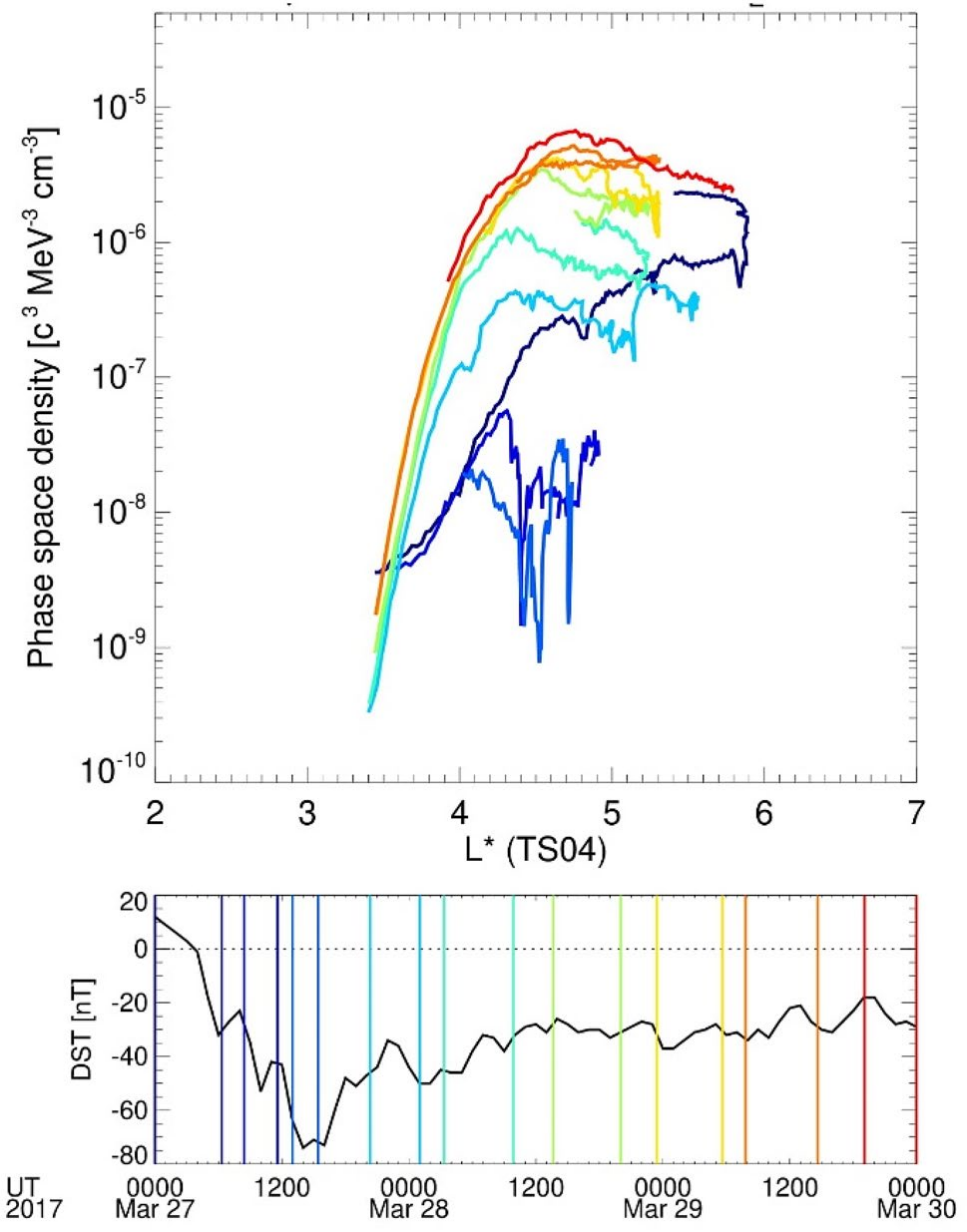
Evolutions of the phase space density of the outer belt during this interval (Top). Phase space density profiles measured by the XEP instrument^15^ on 27, 28, and 29 March 2017. Phase space density, $f$, is in units of (c/cm MeV)^3^, where $c$ is the speed of light. By plotting phase space density at fixed magnetic adiabatic invariants, μ = 1000 MeV/G, and K = 0.2 R_E_G^1/2^. The invariants were calculated using the TS04 magnetic field model^49^. Corresponding energy and pitch angle for μ = 1000 MeV/G, and K = 0.2 R_E_G^1/2^ are shown in Fig. S1 (Bottom). The storm index Dst. Colour lines in top panel correspond to each period in bottom panel.

The chorus waves are primarily responsible for the local acceleration of electrons in the Van Allen radiation belt, leading to a peak in the radial profile of the electron phase space density (PSD). A recent study^27^ reported the simultaneous acceleration and precipitation of MeV electrons owing to chorus waves. In fact, the radial profiles of the PSD at 1000 MeV/G and 0.2 R_E_ G^1/2^ show a growing peak inside the Van Allen radiation belt during the storm, as shown in Fig. 3, suggesting that the chorus waves contribute to the local acceleration of Van Allen belt electrons^28^. Figure S1 shows corresponding energy and pitch angle for 1,000 MeV/G and 0.2 R_E_ G^1/2^ along the satellite orbit.

## Discussion and summary

Previous theoretical studies have suggested that chorus waves propagating towards higher latitudes can also produce electron precipitations over a wide energy range^9,15,29,30^. In this respect, we quantitatively estimate energy spectra of precipitating electron flux caused by chorus waves using a simulation of wave–particle interactions^31^. We injected 2 × 10^6^ test electrons along the field line and solved the equation of motion for each test electron. The equatorial flux distribution from 10 keV to approximately 4 MeV near the loss cone was determined to match that observed by Arase. We also computed the propagation of chorus waves by considering the Arase observed frequency spectrum. The wave amplitude used in the simulation was 80 pT as an average during this interval, while the observed wave amplitude varied with time during this interval. We assumed that the plasma density observed by Arase remained constant along the magnetic field line and that the chorus waves were confined at latitudes below 40°, in agreement with statistical studies^32^.

**Figure 4:**
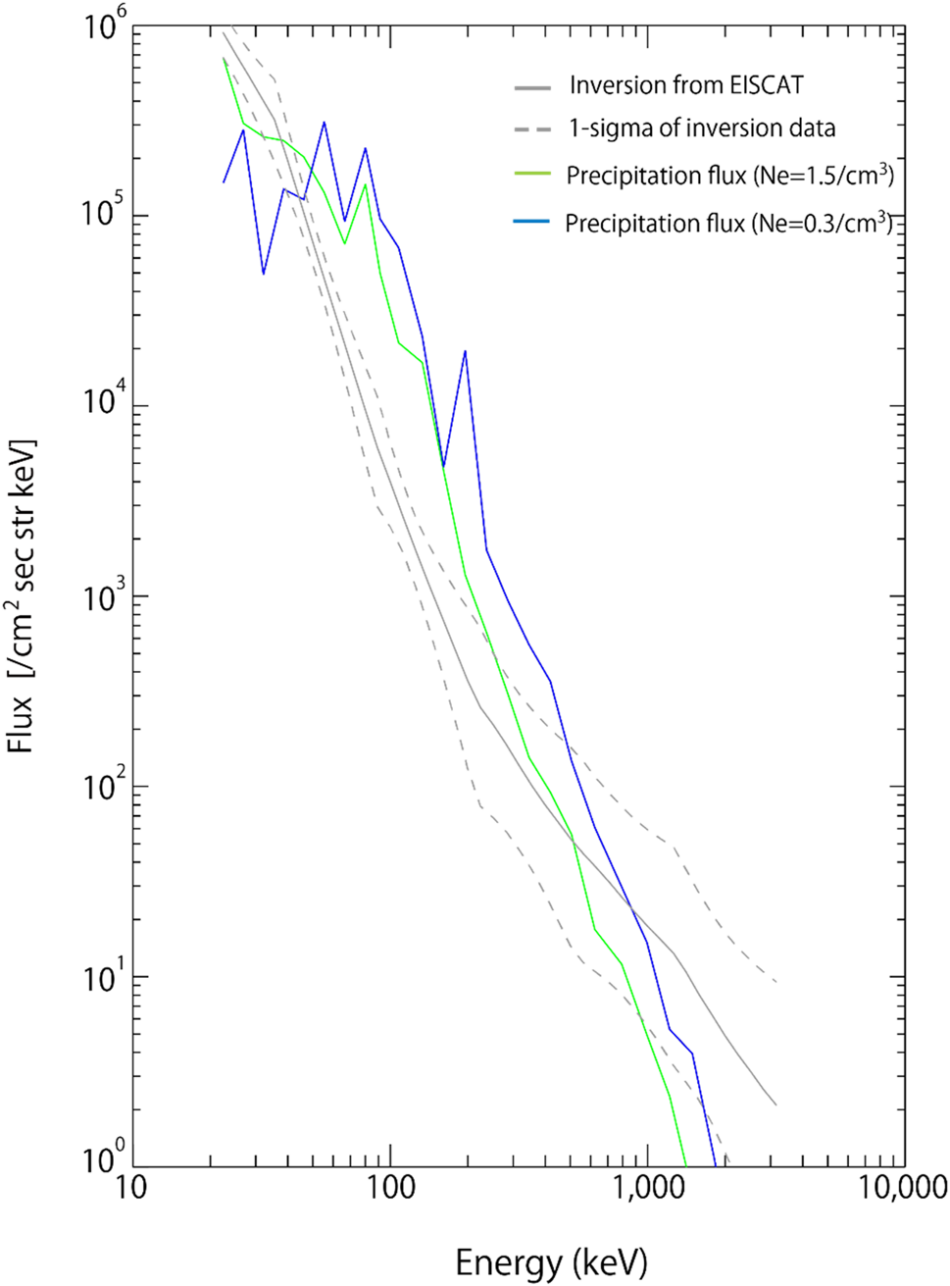
Comparison of wave-particle interaction simulation with EISCAT observations. Energy spectrum of precipitating electrons. The dark solid line indicates the energy spectrum obtained from EISCAT measurements using the inversion method^14^. The black dotted line indicates the 1-sigma errors. The blue and green solid lines indicate the energy spectrum obtained from the computer simulation^21^ of wave-particle interactions for an ambient density of 0.3/cm^3^ and 1.5/cm^3^, respectively.

Considering the uncertainty in the observed plasma density, we simulated two cases, as shown in Fig. 4, for 0.3/cm^3^ (blue solid line) and 1.5/cm^3^ (green solid line). The figure illustrates the estimated precipitation flux at the ionospheric altitudes and the electron spectrum derived from the inversion calculation of the EISCAT data. The dot-dashed lines indicate the 1-sigma error of the energy spectrum derived from EISCAT. During this interval, the chorus wave intensity varies with time, and intense chorus waves exceeding 200 pT in amplitude are often observed. Moreover, the electron flux near the loss cone also varies with time; therefore, the energy spectrum from the simulation should exhibit temporal variation. The consistency between the simulation and the inversion calculation of the EISCAT data indicates that the observed chorus waves do cause the MeV electron precipitations during this interval. There are several discrepancies between the simulation and the EISCAT data. For example, the simulated flux of 30–80 keV electrons is larger than that in the EISCAT data. The simulation assumes the uniform plasma density along the field-line, wave normal angles, which change the resonance conditions, scattering rate, and the propagation latitudes. More accurate parameters of waves and electron flux are essential for future comparisons with the EISCAT data. Electro-Magnetic Ion Cyclotron (EMIC) waves can also cause MeV electron scattering. However, during this interval, the Arase spacecraft measurements showed no evidence of EMIC waves; we can therefore focus on interactions with chorus waves^33^.

**Figure 5:**
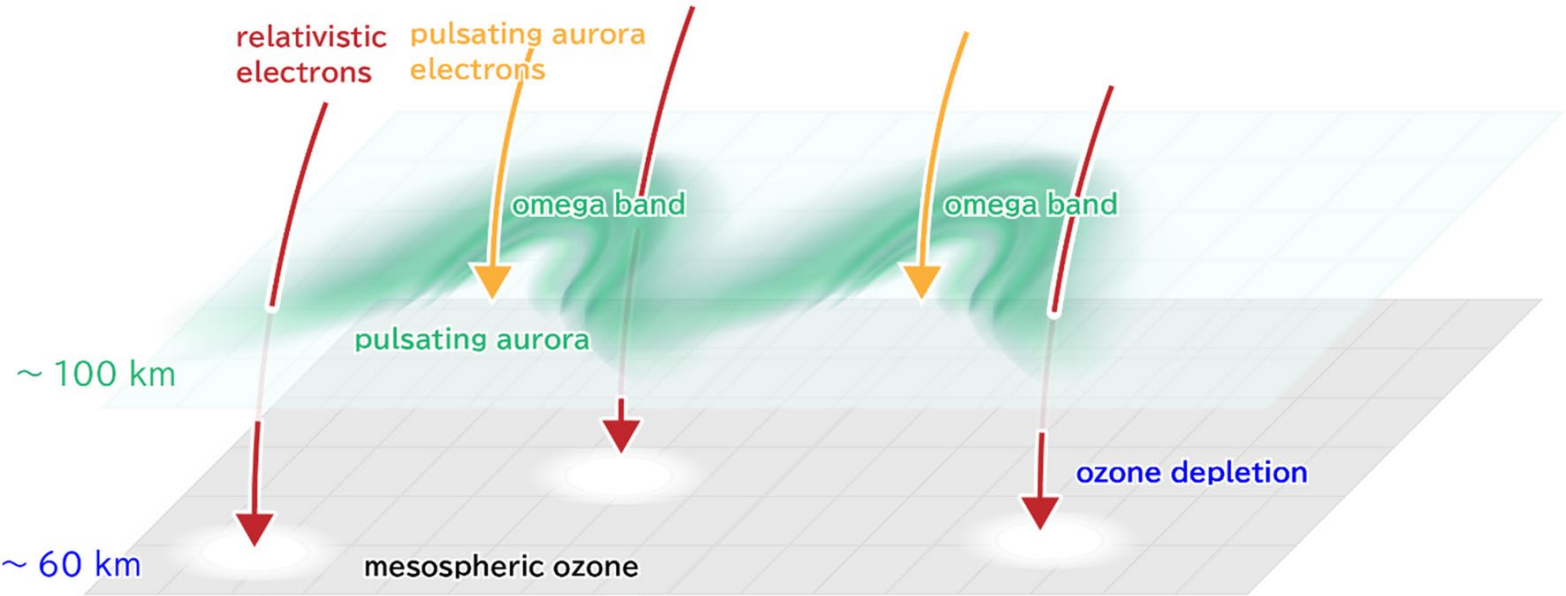
Schematic figure about simultaneous precipitations of tens keV electrons and relativistic electron precipitations. Schematic figure shows tens of keV electrons that cause pulsating aurora near the omega bands at the lower thermosphere. Simultaneously, sub-relativistic/relativistic electrons precipitate further into the mesospheric altitudes, causing significant ozone depletion. (Adobe Illustrator cc 2019. https://www.adobe.com//products/illustrator.html).

Figure 5 shows a schematic diagram illustrating the simultaneous precipitation of 10 s of keV electrons, which reach the lower thermosphere, and 100 s of keV to MeV electrons penetrating even deeper into the mesospheric altitudes. They rush toward the Earth along the same field lines: the former brightens PsA, while the latter causes a local concentration change in mesospheric O_3_, as demonstrated by this study. In the upper mesosphere, the impact is similar to the quantitatively well-known effect of solar proton events^19^. Since PsA occur more frequently (almost daily) than well-known effect of solar proton events^20^, last much longer than our simulated single event, and often extend over large areas^3^, we expect the consequent variations in the O_3_ destruction to be significant. Previous studies^34,35^ frequently observed PsA events with a duration of 9 h or even longer, suggesting that mesospheric O_3_ depletion occurs in a wide magnetic local time. Therefore, the PsA effect must be considered when investigating the long-term composition variations of the middle and upper atmosphere, which manifests the multidisciplinary nature of the interaction between these atmospheric layers and the magnetosphere. In the future, our calculated PsA-driven O_3_ variations should be verified by conducting observations with, e.g., ground-based millimeter-wave spectroscopic radiometers^36^. In comparisons with satellite-based observations statistical methods can also be used to reveal both the short and longer-term ozone responses to PsA forcing^37^.

## Experimental methods

### Test-particle simulation

We used the geospace environment modelling system for integrated studies‐radiation belt with wave‐particle interaction module test particle simulation^38^, which simulates the wave‐particle interaction process between lower band chorus propagating along the field line and the bouncing electrons. The number of test particle is 2 × 10^6^, which are distributed from 10 keV to 3 MeV at the equatorial pitch angle range from 3° to 30°. The simulation estimates the temporal variation of the energy of the precipitating electrons at an altitude of 100 km. The electron momentum changes associated with the wave‐particle interaction are given by the following equation of motion:

$$\frac{d}{{dt}}p_{e} = q\left( {\delta E + v_{e} \times \left( {B + \delta B} \right)} \right)$$

where $v_{e} = p_{e} /m_{e} \gamma$ is the electron velocity, $B$ is the background magnetic field vector, $p_{e}$ is the electron momentum, $q$ is the charge of an electron, $m_{e}$ is the electron rest mass, γ is the Lorentz factor, and δ*E* and δ*B* are the electric and magnetic field perturbations that satisfy the dispersion relation of the parallel propagating whistler mode wave. When electrons interact with the waves, the equation of motion is numerically solved with the time step δ*t* during $\Delta t$, where $\delta t$ is chosen to resolve the gyromotion and $\Delta t$ is the time step chosen to solve the adiabatic guiding center motion. After calculation of the momentum change in $\Delta t$, the first adiabatic invariant of the electron at $t~ + ~\Delta t$ is calculated using the background magnetic field intensity at the electron position. Simultaneously with the scattering process, the electron guiding center position is advanced, in keeping with the first and second adiabatic invariants. The perturbation components are the same as that reported in previous study^29^. The minimum frequency and the maximum frequencies are 0.3 $f_{{ceq}}$, where $f_{{ceq}}$ is the electron cyclotron frequency at the magnetic equator and 0.5 $f_{{ceq}}$, respectively. The duration of each element is 100 ms and sweep rate of each element is 2.0 $f_{{ceq}}$. The bursts appear every 5 s, and three rising tone elements are embedded in each burst. The repeat frequency of the rising tone elements is 3 Hz, which is a typical modulation frequency of the internal modulation of PsA^15^.

### Ion-chemistry simulation at the upper/middle atmosphere

We used the Sodankylä Ion and Neutral Chemistry (SIC) model that is a 1-D atmospheric model which solves for concentration of 16 minor neutral species (including HOx, NOx, and Ox) and 72 ion species at altitudes between 20 to 150 km^20^. The model includes 389 ion-neutral and neutral–neutral reactions, 2523 ion-ion and electron–ion recombination reactions, and molecular and eddy diffusion. The background neutral atmosphere (for example, N_2_, O_2_, and temperature) are calculated using the empirical NRLMSISE-00 model which depends on daily average values of solar F10.7 radio flux and geomagnetic activity through the Ap index. The daily average solar spectrum is calculated using the SOLAR2000 empirical solar irradiance model. In addition to solar radiation, SIC can be driven by electron precipitations, which has been used in this study. A detailed description of the SIC model is given in^20^.

### Estimation of energetic electron spectrum from EISCAT observations

We used the inversion method by utilizing a Metropolis–Hastings Markov Chain Monte Carlo method (MCMC) and the SIC model as a forward theory of the ionospheric response to the precipitations. The detail procedure of the inversion is described in^18^.

## Supplementary Information

Supplementary Information.

## Data Availability

The Arase data is available from the ERG Science Centre operated by ISAS/JAXA and ISEE/Nagoya University (https://ergsc.isee.nagoya-u.ac.jp/data_info/index.shtml.en)^39^. The present data analysed the MEP e L2 v01_02^40,41^, HEP L2 v03_01^42,43^, XEP L2 v01_00^44^, PWE/OFA L2 v02_01^45^, MGF L2 v03_04^46^, Orbit L3 v01 data^47^. The ground-based optical data from Kiruna, Sweden, used in this paper were obtained through the database of optical instruments at the National Institute of Polar Research, Japan (http://pc115.seg20.nipr.ac.jp/www/opt/). The EISCAT data used in this paper were obtained through the database of EISCAT at the National Institute of Polar Research, Japan (http://polaris.nipr.ac.jp/~eiscat/eiscatdata/). The Dst index data and the AL index were provided by the World Data Centre for Geomagnetism, Kyoto (http://wdc.kugi.kyoto-u.ac.jp/wdc/Sec3.html)^48^.
