# Supplementary material for: Penetration of MeV electrons into the mesosphere accompanying pulsating aurorae
Source: Sci Rep. 2021 Jul 13;11:13724. doi: 10.1038/s41598-021-92611-3 (PMC8277844; doi:10.1038/s41598-021-92611-3)
Supplement: Supplementary file 1 — Supplementary Information. [file 41598_2021_92611_MOESM1_ESM.docx]

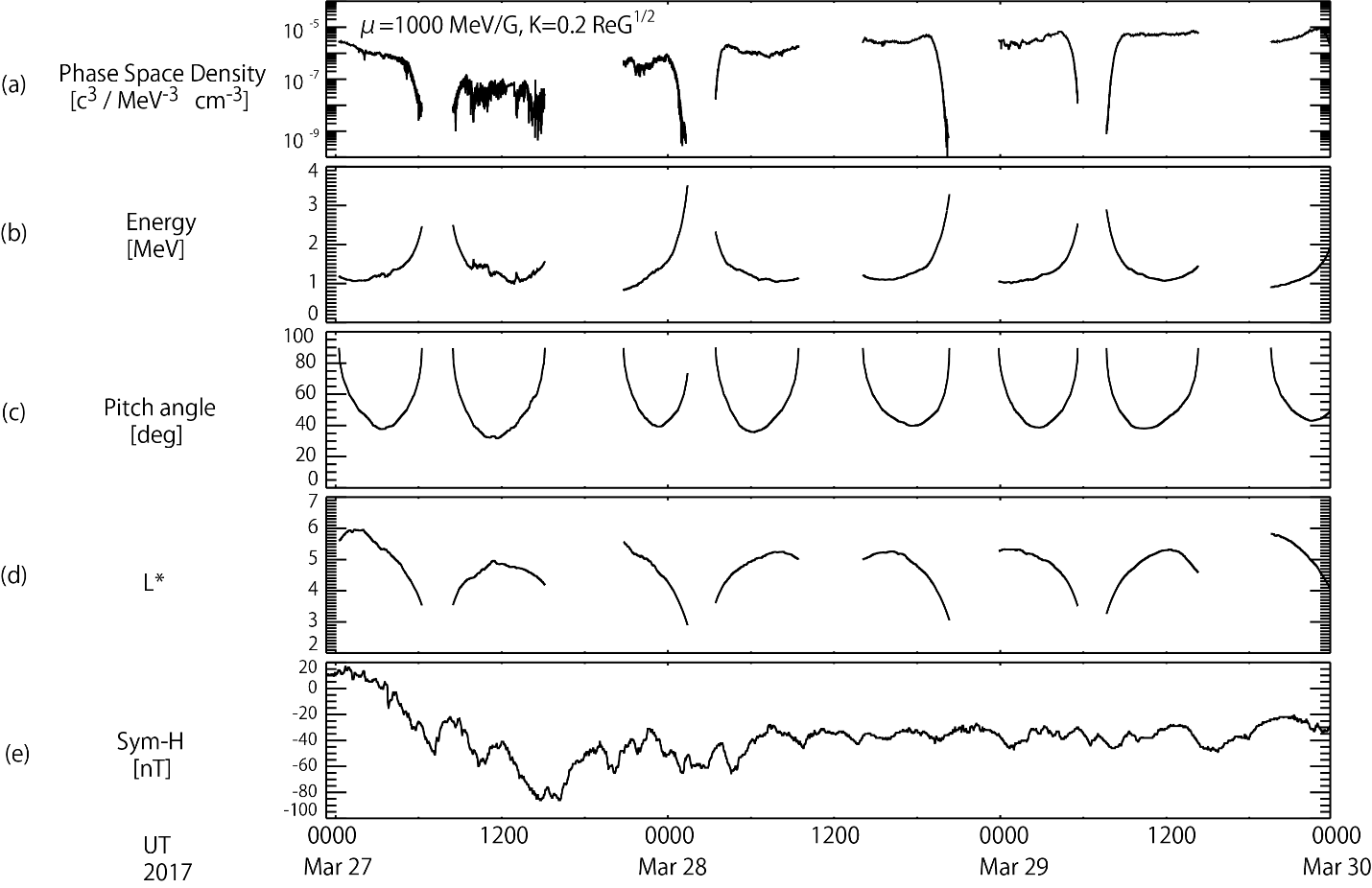


**Figure S1: Time variations of the phase space density (**μ**=1000 MeV/G and K=0.2 Re G^1/2^) along the satellite orbit.**

(a) Phase space density of μ=1000 MeV/G, and K=0.2 Re G^1/2^ along the satellite orbit derived by Arase/XEP measurements. Corresponding energy and local pitch angle for these adiabatic invariants are shown in (b) and (c), respectively. The phase space density is derived from the interpolation about energy and pitch angle using the observed differential flux. (d) L* of the Arase spacecraft. (e) Sym-H index.

(Adobe Illustrator cc 2019. https://www.adobe.com//products/illustrator.html)
